# RNAa Is Conserved in Mammalian Cells

**DOI:** 10.1371/journal.pone.0008848

**Published:** 2010-01-22

**Authors:** Vera Huang, Yi Qin, Ji Wang, Xiaoling Wang, Robert F. Place, Guiting Lin, Tom F. Lue, Long-Cheng Li

**Affiliations:** 1 Helen-Diller Comprehensive Cancer Center, University of California San Francisco, San Francisco, California, United States of America; 2 Department of Urology, University of California San Francisco, San Francisco, California, United States of America; University of Hong Kong, Hong Kong

## Abstract

**Background:**

RNA activation (RNAa) is a newly discovered mechanism of gene *activation* triggered by small double-stranded RNAs termed ‘small activating RNAs’ (saRNAs). Thus far, RNAa has only been demonstrated in human cells and is unclear whether it is conserved in other mammals.

**Methodology/Principal Findings:**

In the present study, we evaluated RNAa in cells derived from four mammalian species including nonhuman primates (African green monkey and chimpanzee), mouse, and rat. Previously, we identified saRNAs leading to the activation of E-cadherin, p21, and VEGF in human cells. As the targeted sequences are highly conserved in primates, transfection of each human saRNA into African green monkey (COS1) and chimpanzee (WES) cells also resulted in induction of the intended gene. Additional saRNAs targeting clinically relevant genes including p53, PAR4, WT1, RB1, p27, NKX3-1, VDR, IL2, and pS2 were also designed and transfected into COS1 and WES cells. Of the nine genes, p53, PAR4, WT1, and NKX3-1 were induced by their corresponding saRNAs. We further extended our analysis of RNAa into rodent cell types. We identified two saRNAs that induced the expression of mouse Cyclin B1 in NIH/3T3 and TRAMP C1 cells, which led to increased phosphorylation of histone H3, a downstream marker for chromosome condensation and entry into mitosis. We also identified two saRNAs that activated the expression of CXCR4 in primary rat adipose–derived stem cells.

**Conclusions/Significance:**

This study demonstrates that RNAa exists in mammalian species other than human. Our findings also suggest that nonhuman primate disease models may have clinical applicability for validating RNAa-based drugs.

## Introduction

RNA activation (RNAa) is a newly discovered mechanism of gene *activation* directed by small double-stranded RNAs (dsRNAs) [1], [2], [3]. These dsRNAs, termed ‘small activating RNAs’ (saRNAs), exert an effect opposite to that of RNA-interference (RNAi). Several models of RNAa have been reported or proposed including transcriptional activation by targeting promoter-specific sequences [1], [3], [4], [5] and/or gene antisense transcripts [6], [7] leading to changes in chromatin structure at the targeted gene. Small RNAs have also been shown to enhance post-transcriptional gene expression by directly promoting translation [8] or antagonizing miRNA target recognition [9]. Taken together, there are likely multiple mechanisms in which small RNAs can mediate gene induction. Regardless, what is becoming clear is that RNAa has potential to be exploited to induce the expression of a variety of genes in human cells.

RNAa is generally potent and long-lasting [1], [2] making it a promising therapeutic strategy for diseases that can be corrected by stimulating gene expression. Ideally, RNAa can be applied as a cancer treatment to re-activate tumor suppressor or pro-apoptotic genes that are otherwise not targetable by current therapeutic strategies. Although small animals such as mice and rats are frequently used to test small molecule drugs, they may not be suitable for testing RNA-based therapies. Because human and rodent genetic sequences can diverge, a functional small RNA in rodents may not be valid in humans, especially for those that target gene promoter sequences. In contrast, non-human primates share almost identical genome sequences with human and are more ideal for testing genetic therapies. In fact, non-human primate disease models are often used to test preclinical safety and efficacy of vector-based gene therapy [10] and for testing systematic delivery of RNAi-based therapeutics [11], [12], [13].

So far, RNAa has only been demonstrated in human cells and is unknown whether it is conserved in other mammals. In the present study, we replicate and identify new examples of RNAa in non-human primate, mouse, and rat cells. We show that saRNAs derived from human sequences have RNAa activity in non-human primate cells. This suggests non-human primate disease models may have clinical relevance in validating RNAa-based drugs. We also demonstrate RNAa in other mammalian species by testing saRNAs derived from mouse and rat sequence in rodent cells. Our findings reveal that RNAa is prevalent in mammalian species other than human with most of the genes tested being readily activated by saRNAs.

## Results

**Figure 1:**
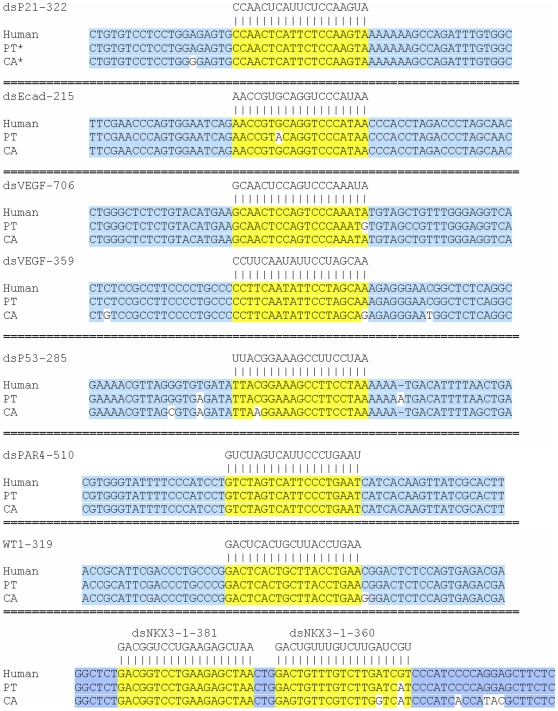
Primate saRNA sequences and target alignments. Genomic DNA was isolated from WES and COS1 cells. Gene promoter regions surrounding saRNA target sites were sequenced using promoter-specific primers. Indicated are the saRNA sequences aligned to human, *Pan troglodytes* (PT; chimpanzee), and *Cercopithecus aethiops* (CA; African green monkey) promoter sequences. Target sites are highlighted in yellow, while non-target sequence is highlighted in blue. Genomic sequences divergent from human are not highlighted. The numerical designation within the name of each saRNA denotes the target location on each gene promoter relative to the transcription start site of the human gene.

**Figure 2:**
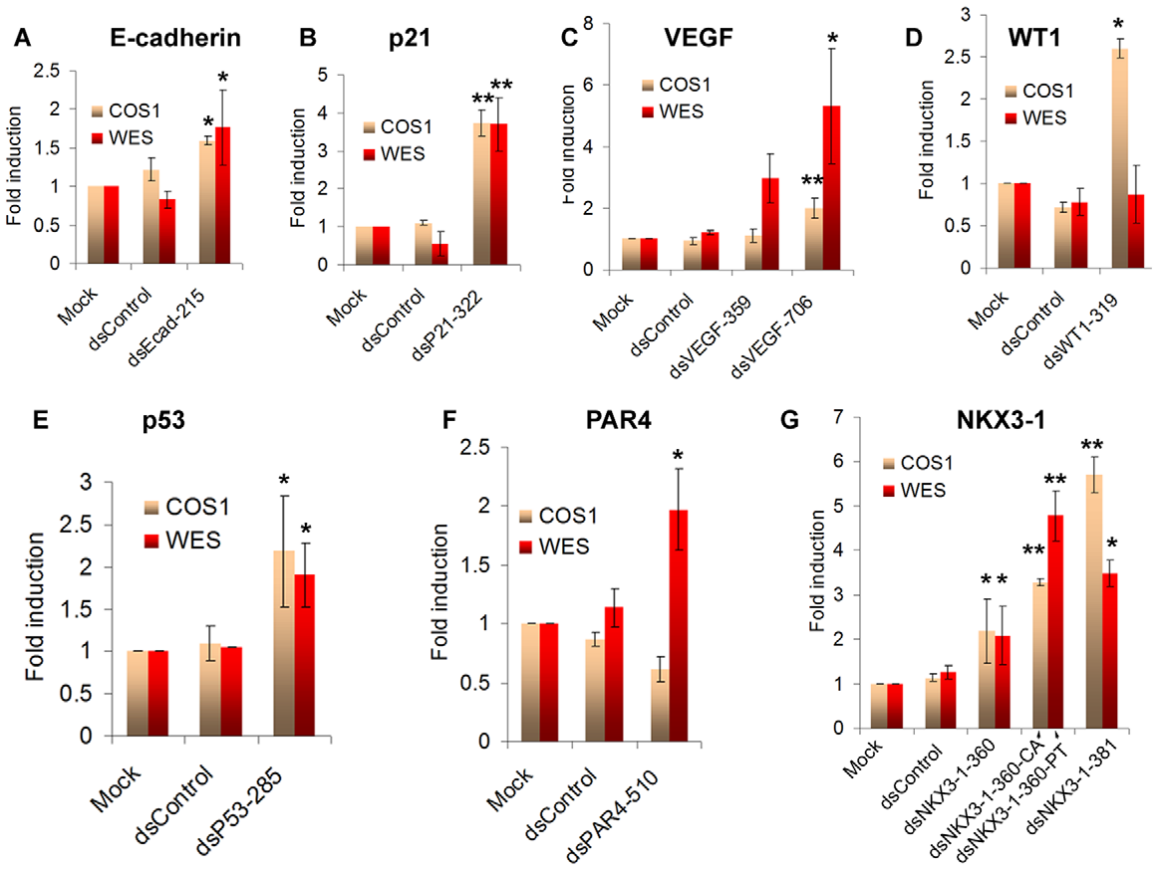
RNA activation of different genes in non-human primate cells. **A–G**. COS1 and WES cells were transfected with 25 nM of the indicated saRNAs for 3–5 days. Mock samples were transfected in the absence of saRNA. mRNA expression levels were analyzed by real-time PCR and normalized to β-actin. Expression levels were measured as fold induction relative to mock transfections. The results are represented as mean ± SEM of three independent experiments. Species-specific saRNAs were designed to perfectly complement their respective chimpanzee (dsNKX3-1-360-PT) and AGM (dsNKX3-1-360-CA) targeted sequences in WES and COS1 cells, respectively. Statistical significance is indicated (* *p*<0.05, ** *p*<0.01) as compared to mock treatments.

We previously identified saRNAs that activated that expression of E-cadherin, p21, and VEGF in human cells [1]. Sequence alignment utilizing public genome databases revealed that the intended targeted sites for each human saRNA in primates (*e.g.* Chimpanzee, Orangutan, and Rhesus monkey) were all highly-conserved with human (data not shown). Conservation was confirmed by sequencing DNA amplified from non-human primate COS1 (African green monkey, AGM) and WES (chimpanzee) cell lines (Fig. 1, GenBank accession numbers for the resulted sequences are listed in Materials and Methods). Our results also revealed subtle species-specific variations in sequence between human, AGM, and chimpanzee excluding the possibility of potential contamination of non-human primate cell lines with human cells (Fig. 1). In order to replicate the phenomenon of RNAa in non-human primate cells, we transfected both cell lines with human saRNA targeting the E-cadherin promoter at position −215 (dsEcad-215) or p21 promoter at position −322 (dsP21–322) relative to the transcription start sites (TSS). Compared to control treatments, dsEcad-215 and dsP21–322 caused a ∼1.6- (*p*<0.05) and ∼3.7-fold (*p*<0.05) increase in E-cadherin and p21 expression levels, respectively, in both cell lines (Fig. 2A and B). Similar to what we observed in human cells [1], activation of either E-cadherin or p21 in COS1 and WES cells significantly inhibited cell proliferation and survival reflecting their roles as tumor suppressor genes (Fig. S1). We also tested previously identified human VEGF saRNA dsVEGF-706 and newly designed dsVEGF-359 in both primate cell lines. In WES cells, dsVEGF-359 and dsVEGF-706 induced VEGF mRNA expression by ∼3.0- (*p* = 0.2) and ∼5.3-fold (*p*<0.05), respectively (Fig. 2C). In COS1 cells, dsVEGF-706 significantly induced VEGF mRNA expression by ∼2-fold (*p*<0.01, Fig. 2C). ELISA confirmed that the secreted form of VEGF (VEGF_165_) was induced following saRNA treatment (Fig. S2).

To expand the list of known RNAa-responsive genes in primate cells, we used a set of design rules (Materials and Methods) to select saRNA targets for the following clinically relevant genes: p53, PAR4, WT1, RB1, p27, NKX3-1, VDR, IL2, and pS2. Genome sequence alignment revealed that these saRNA target sites are well conserved between human and primates (Fig. 1 and data not shown). Of the nine genes, p53, PAR4, WT1 and NKX3-1 were induced ≥1.5-fold by their corresponding saRNA in at least one of the two non-human primate cell lines (Fig. 2D–G).

To activate NKX3-1 expression, two saRNAs were designed that targeted the human NKX3-1 promoter at positions −360 (dsNKX3-1-360) and −381 (dsNKX3-1-381) relative to the TSS. The human dsNKX3-1-381 target sequence perfectly matched those in the NKX3-1 promoter of chimpanzee and AGM, while the dsNKX3-1-360 target site shared 94.7% and 84.2% sequence identity with its cognates in chimpanzee and AGM, respectively (Fig. 1). Compared to controls, dsNKX3-1-381 robustly induced NKX3-1 mRNA expression in both WES (∼3.5-fold, *p*<0.05) and COS1 (∼5.7-fold, *p*<0.01) cells (Fig. 2G), as well as displayed altered morphology and decreased cell density characteristic of the predicted tumor suppressor function of NKX3-1 [14] (Fig. S3). Interestingly, despite having non-perfect homology, dsNKX3-1-360 was still capable of inducing NKX3-1 mRNA expression by ∼2-fold in both cell lines (Fig. 2G, *p*<0.05). To evaluate the specificity of the saRNA (*i.e.* whether improved sequence complementarity would enhance RNAa activity), we designed species-specific NKX3-1 saRNAs that perfectly matched their respective chimpanzee (dsNKX3-1-360-PT) and AGM (dsNKX3-1-360-CA) targeted sequences. Both species-specific saRNAs were found to have stronger RNAa activity compared to human dsNKX3-1-360; NKX3-1 mRNA expression was increased by ∼4.8- and ∼3.3-fold in WES and COS1 cells, respectively (*p*<0.01, Fig. 2G). These results suggest that NKX3-1 activation is sequence-dependent and RNAa in non-human primates can tolerate mismatches in a manner analogous to microRNA target recognition.

**Figure 3:**
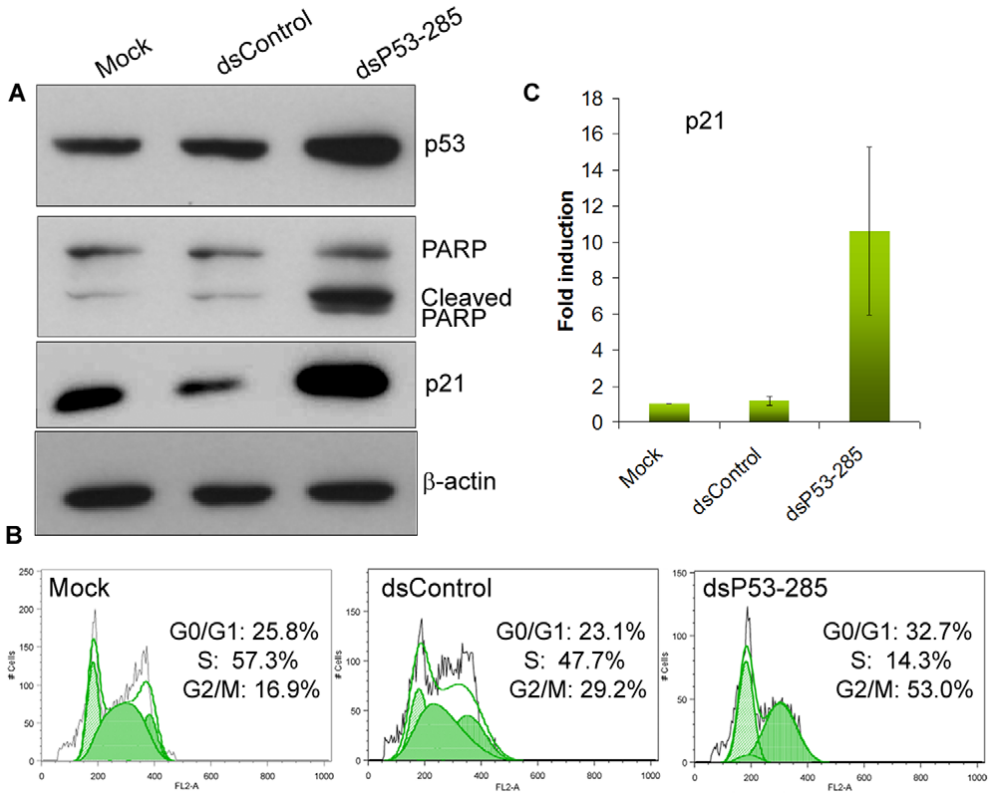
Activation of p53 by saRNA causes cell cycle arrest and induction of p21 in WES cells. **A**. WES cells were transfected with 25 nM of the indicated saRNAs for 5 days. Protein levels of p53, PARP and p21 were detected by immunoblot analysis. β-actin served as a loading control. **B**. WES cells were transfected as in A. Cells were collected, stained with propidium iodide, and processed for analysis by flow cytometry to measure DNA content. Shown are examples of resulting FL2A histograms following analysis with FlowJo software. Cell populations distributed in G0/G1, S, and G2/M phases of the cell cycle are indicated. **C**. WES cells were transfected with 25 nM of the indicated saRNAs for 72 hrs. p21 mRNA expression was analyzed by real-time PCR. The results represent mean ± SEM of two independent experiments and are plotted as fold induction relative to mock transfections.

The p53 saRNA targeted sequence position −285 (dsP53–285) relative to the TSS in the human p53 promoter. The −285 site is comprised of a repetitive region unique to the p53 promoter that is almost identical amongst human, chimpanzee, and AGM (Fig. 1). In WES cells, activation of p53 by dsP53–285 resulted in PARP cleavage indicative of caspase-dependent apoptosis; a consequence of p53 overexpression [15] (Fig. 3A). To evaluate cell cycle distribution, DNA content was analyzed by flow cytometry in cells stained with propidium iodide. Transfection of dsP53–285 caused a significant increase in G1/G0 and G2/M populations with a concurrent decline in S populations as compared to control treatments (Fig. 3B). Cell cycle arrest is associated with p53 induction and activation of downstream cell cycle inhibitory protein p21 [16]. Consistent with this observation, p21 was also induced by over 10-fold in dsP53-285-transfected cells (Fig. 3C).

**Figure 4:**
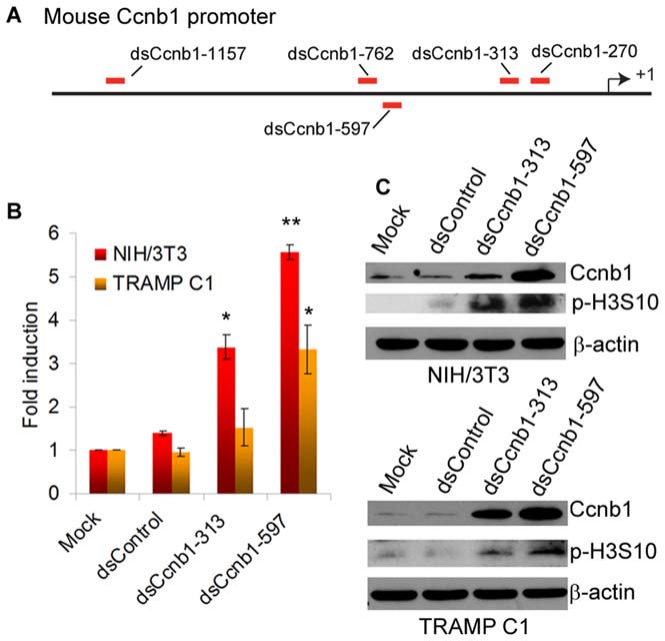
Ccnb1 activation in mouse cells by promoter-targeting saRNAs. **A**. A schematic representation of the mouse Ccnb1 promoter. Indicated is the transcription start site (+1) and locations of the target sites for each putative saRNA. **B**. saRNAs were transfected into mouse NIH/3T3 and TRAMP C1 cells at 50 nM concentrations for 5 days. Ccnb1 mRNA levels were determined by real-time RT-PCR and normalized to β-actin levels. Results are mean ± SEM of 3 independent experiments. Statistical significance is indicated (* *p*<0.05, ** *p*<0.01) as compared to mock treatments. **C**. NIH/3T3 and TRAMP C1 cells were transfected as in B. Levels of Ccnb1 protein and phospho-histone H3 at serine 10 (p-H3S10) were determined by immunoblot analysis. Representative immunoblots are shown from 3 independent experiments.

We further extended our analysis of RNAa into mouse cells by selecting Cyclin B1 (Ccnb1) as a candidate gene. Using the same design rules, five saRNA targets were chosen on the mouse Ccnb1 promoter between −1200 to −270 bp relative to the TSS (Fig. 4A). We transfected the corresponding saRNAs into NIH/3T3 and transgenic adenocarcinoma mouse prostate (TRAMP) C1 cells and identified two saRNAs (dsCcnb1–313 and dsCcnb1–597) that elevated Ccnb1 mRNA expression levels. In NIH/3T3 cells, dsCcnb1–313 and dsCcnb1–597 induced Ccnb1 levels by ∼5.5- (*p*<0.01) and ∼3.3-fold (*p*<0.05), respectively; whereas dsCcnb1–597 induced Ccnb1 levels by ∼3.2-fold (*p*<0.05) in TRAMP C1 cells (Fig. 4B). Because Ccnb1 promotes entry into mitosis, induction of Ccnb1 by dsCcnb1–313 or dsCcnb1–597 also increased the phosphorylation of histone H3 at serine 10 (p-H3S10) correlating with chromosome condensation during mitosis [17] (Fig. 4C).

**Figure 5:**
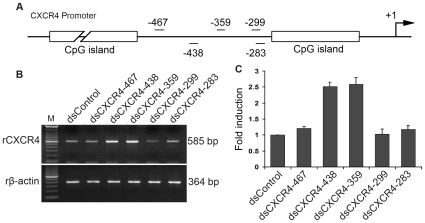
RNAa-mediated induction of CXCR4 in primary rat stem cells. **A**. A schematic representation of the rat CXCR4 promoter. Indicated are the CpG islands, transcription start site (+1), and locations of the target sites corresponding to each of the five putative saRNAs. **B**. Each saRNA was transfected at 50 nM concentrations into rADSCs for 4 days. CXCR4 and β-actin mRNA expression levels were assessed by standard RT-PCR. β-actin served as a loading control. **C**. Expression of CXCR4 was quantified by densitometry and adjusted to β-actin levels. Results are mean ± SEM of 3 independent experiments.

To explore RNAa in rat cells, we designed five saRNAs targeting sequences between −500 and −250 bp relative to the TSS in the promoter of rat chemokine receptor CXCR4 (Fig. 5A). Primary rat adipose-derived stem cells (rADSCs) were transfected with each duplex and CXCR4 expression levels were evaluated by RT-PCR. Remarkably, two saRNAs (dsCXCR4–359 and dsCXCR4–438) were identified that activated CXCR4 expression (Fig. 5B). Compared to control treatments, dsCXCR4–359 and dsCXCR4–438 both induced CXCR4 levels by ∼2.5-fold in rADSCs (Fig. 5C).

## Discussion

RNA activation (RNAa) is a newly discovered mechanism of small RNA-directed gene activation originally identified in human cell lines [1], [2], [3]. It offers similar benefits as RNAi by utilizing small dsRNAs, while representing a new method for gene overexpression. As the momentum of RNAa increases within the scientific community, it is important to determine if this phenomenon is conserved in other mammalian species. Within this study, we replicate and identify new examples of RNAa in non-human primates, mouse, and rat cells. This not only demonstrates conservation of RNAa, but also reveals its potential application in animal studies other than human. In fact, during the preparation of this manuscript, it was shown that lentiviral-based delivery of shRNAs could activate VEGF expression by targeting promoter sequences in an ischemic mouse model [18]. Furthermore, our findings suggest that non-human primate disease models may have clinical application for validating RNAa-based therapeutics. Because primates share almost identical genome sequences with human, functional saRNAs may be conserved with equal efficacy between non-human primates and humans.

RNAa offers a promising new therapeutic strategy for disease that can be corrected by stimulating gene expression. Most of the genes targeted within this study have clinical relevance in regulating tumor cell growth. For instance, restoration of p53 function has recently been shown to cause dramatic tumor regression in mice [19], [20], [21]. As such, the feasibility of activating p53 by RNAa may have implications in cancer treatment. Similarly, the tumor suppressive function of NKX3-1 in prostate cancer may also offer a RNAa-based target for prostate cancer therapy.

Application of RNAa is not limited to cancer therapeutics. It has potential to serve as a substitute for vector-based overexpression in a variety of systems. For instance, we demonstrate activation of CXCR4 in primary rADSCs. CXCR4 plays an important role in migration, engraftment, and proliferation of stem cells [22], [23], [24]. As such, RNAa may be useful in promoting stem cell phenotype by targeting genes involved in reprogramming. Furthermore, activation of CXCR in primary rat cells suggests RNAa has relevant use in primary cell systems, as well.

Design of saRNAs has largely been a hit-or-miss process due to a lack of complete understanding of the mechanism(s) and the heterogeneous nature of different target regions with regard to their impact on gene transcription. However, our design rules have been met with some success. In the case of mouse Ccnb1 and rat CXCR4, 2 out of the 5 saRNAs tested were capable of activating target gene expression. Similarly, 4 out of the 9 genes screened in non-human primate cells were found to be susceptible to RNAa in at least one cell line. For the five genes we failed to activate by RNAa (i.e. RB1, p27, VDR, IL2, and pS2), only one saRNA was tested for each promoter. If more targets were screened, it may be possible that these genes would be responsive to RNAa. However, it is important to note that not all genes may be susceptible to RNAa. For instance, genes silenced by DNA methylation may confer resistance to RNAa [1]. For this reason, basal expression was evaluated to confirm that the targeted genes were not completely silenced. Regardless, RNAa offers a new approach to enhance endogenous gene expression, which may be manipulated to target a variety of genes. As such, RNAa has potential to function as a surrogate tool for vector-based gene overexpression systems.

## Materials and Methods

### Cell Culture and Transfection

African green monkey (*Cercopithecus aethiops*) COS1 kidney fibroblast-like cells, Chimpanzee (*Pan troglodytes*) skin fibroblast cells (WES), mouse NIH/3T3 cells were obtained from American Type Culture Collection (Rockville, MD) and the UCSF Cell Culture Facility. TRAMP C1 cells were kindly provided by Dr. Marcella Fasso with permission from Dr. Norman Greenberg. COS1 and WES cells were grown in Dulbecco's Modified Eagle's Medium with 10% (v/v) fetal bovine serum (FBS), 100 U/ml penicillin, 100 µg/ml streptomycin, and 2 mM L-glutamine. NIH/3T3 cells were grown in Dulbecco's Modified Eagle's Medium with 10% (v/v) bovine calf serum, 100 U/ml penicillin, 100 µg/ml streptomycin, and 2 mM L-glutamine. TRAMP C1 cells were grown as previously described [25]. Immediately before transfection, cells were trypsinized, diluted with growth medium without antibiotics, and seeded in 6-well plates (∼2.5×10^5^ cells for COS1 and WES cells and ∼3×10^4^ cells for NIH/3T3 and TRAMP C1 cells). Transfection of saRNA was carried out using Lipofectamine RNAiMax or 2000 (Invitrogen, Carlsbad, CA) according to the reverse transfection protocol provided with the product. The cells were harvested 3–5 days following transfection for analysis.

### Rat Adipose–Derived Stem Cell (rADSC) Isolation and Culture

Isolation and culture of rADSCs were performed as previously described [26]. Briefly, rat adipose tissue was rinsed with PBS containing 1% antibiotics, minced into small pieces, and incubated in a solution of 0.075% collagenase Type IA (Sigma-Aldrich, St. Louis, MO) for 1 h at 37°C. The upper insoluble lipid layer was removed and the remaining liquid was centrifuged at 200×g for 10 min at room temperature. The pellet containing rADSCs was treated with 160 mM NH_4_Cl for 10 minutes to lyse red blood cells. The remaining cells were suspended in Dulbecco's modified Eagle's medium (DMEM) supplemented with 10% (v/v) FBS and plated at a density of 1×10^6^ cells in 10 cm dishes. After reaching ∼80% confluence, cells were split and cultured in DMEM supplemented with 10% (v/v) FBS, 1% nonessential amino acid, 100 U/ml penicillin, 100 µg/ml streptomycin, 0.025 mg/ml fungizone, and 110 mg/ml sodium pyruvate.

### dsRNA Design

Promoter sequences for all primates were retrieved from Ensembl genome databases (www.ensembl.org) and analyzed against several genome and sequence databases including dbTSS (Database of Transcriptional Start Sites, http://dbtss.hgc.jp), AceView (http://www.ncbi.nlm.nih.gov/IEB/Research/Acembly) and UCSC Genome Browser (http://genome.ucsc.edu) to determine transcription start sites. Sequence alignments among all primates were provided by the Ensembl database. Human promoter sequences were used as the standard for saRNA design in non-human primates. Promoter sequence was inputted into an Excel macro template that implemented saRNA design rules derived from our previous and on-going studies [1], [3]. Based on these rules, the program assigned weighted scores to all possible targets and returned a list of targets sorted by sum scores. All tested saRNAs are listed in Table S1. Design rules are listed below.

1. Targets were picked on the sense DNA sequence.
2. The RNA strand complementary to the sense DNA strand was determined to be the guide strand; thus, the 3′ end of the DNA target corresponded to the 5′ end of the guide RNA strand.
3. saRNA targets were selected in a region between approximately −1200 bp to −200 bp upstream of transcription start sites.
4. The size of each target is 19 nt. The resulting saRNA duplexes possessed either dTdT or UU overhangs at the 3′ end of each strand.
5. Targets had a GC content of ∼40–65%.
6. Consecutive nucleotides >4 were avoided.
7. The 3′ end of a target possessed a lower thermodynamic stability than its 5′ end.
8. The 19th position was an “A”.
9. The 18th position was an “A” or “T”, preferably “A”.
10. The 7th position was preferably a “T”.
11. The 20th–23rd positions (flanking the 3′ end of a target) were preferably “A”s or “T”s
12. Sites susceptible to DNA methylation such as CpG islands, CpG sites, and GC-rich regions were avoided because DNA methylation was previously shown to interfere with RNAa activity [1].

### Promoter DNA Sequencing

DNA was extracted from COS1 and WES cells. Primers complementary to genomic sequence (Table S1) were used to amplify regions surrounding saRNA targeted sites in gene promoters. PCR products were purified using a DNA clean-up kit (Zymo Research, Orange, CA) and sequenced in both directions. The resulted DNA sequences were deposited in GenBank with the following accession numbers: GQ982508, GQ982509, GQ982510, GQ982511, GQ982512, GQ982513, GQ982514, GQ982515, GQ982516, GQ982517, GQ982518, GQ982519, GQ982520, and GQ982521.

### RNA Isolation and Analysis of Gene Expression

RNA from rADSCs was isolated by using the High pure RNA isolation Kit (Roche, Indianapolis, IN). RNA from all other cell lines was isolated by using the RNeasy Mini Kit (Qiagen, Valencia, CA). Each RNA sample was treated with RNase-free DNase I (Qiagen) to remove any potential contaminating DNA. One microgram of RNA was used for cDNA synthesis using oligo(dT)_15–18_ primers and M-MLV reverse transcriptase (Promega, Madison, WI). Real-time PCR was performed to quantify gene expression by using gene-specific primer sets in conjunction with Power SYBR Green PCR Master Mix (Applied Biosystems, Foster City, CA). Semi-quantitative RT-PCR was utilized to evaluate rat CXCR4 and β-actin expression. PCR primer sequences are listed in Table S1.

### ELISA Analysis

Cells were transfected with 25 nM VEGF saRNA (dsVEGF-358 or dsVEGF-706) for 72 h. Culture media was then collected and viable cells were counted by trypan blue staining. The relative concentration of secreted VEGF in the tissue culture media was quantified by using the Quantikine Human VEGF Immunoassay ELISA kit (R&D Systems, Minneapolis, MN) according to the manufacturer's instructions. VEGF concentrations were normalized to the number of viable cells in each treatment.

### Immunoblot Analysis

Cells were washed with PBS and lysed in RIPA buffer (50 mM Tris, pH 7.4, 150 mM NaCl, 1% Triton X-100, 0.5% deoxycholate, 0.1% SDS) with protease and phosphatase inhibitor cocktail for 15 min at 4°C. Lysates were clarified by centrifugation and supernatants were collected. Protein concentration was determined by using a BCA protein assay kit (Thermo Scientific, Waltham, MA). Proteins were resolved on SDS-PAGE gels and transferred to nitrocellulose membranes. The resulting blots were blocked with 5% non-fat dry milk and specific proteins were detected with primary antibodies. Blots were subsequently incubated with appropriate HRP-conjugated secondary antibodies and antigen–antibody complexes were visualized by chemiluminescence (Thermo Scientific). Primary antibodies used to detect p53, Ccnb1, PARP, and β-actin protein levels included mouse monoclonal anti-p53 (DO1; CalBiochem, San Diego, CA) at 1∶5000, mouse monoclonal anti-Ccnb1 (V152; Cell signaling) at 1∶1000, rabbit polyclonal anti-PARP (Cell Signaling, Danvers, MA) at 1∶1000, and mouse monoclonal anti-β-actin (Clone AC-15; Sigma) at 1∶3000. Phosphorylation of histone H3 at serine 10 was detected by a rabbit polyclonal anti-phospho-histone H3 (ser10) (Cell Signaling) at 1∶500.

### Clonogenic Survival Assay

Exponentially growing cells were seeded in 12-well plates and reverse transfected with 25 nM saRNA using Lipofectamine RNAiMax. After 24 hrs, the transfected cells were harvested and seeded in 6-well plates at a density of 2000 cells/well. Culture medium without antibiotics was changed every 3 days. Colony formation was analyzed at day 12 by staining cells with crystal violet.

### Analysis of DNA Content by Flow Cytometry

saRNA transfected cells (1×10^6^ cells/ml) were trypsinized and centrifuged at 2500×g at 4°C for 5 min and washed twice in PBS buffer. The pellet was gently resuspended in 200 µl of PBS. The resuspended cells were fixed with 5 ml of 70% cold (−20°C) ethanol on ice for 2 hrs. Before flow cytometry analysis, cells were washed twice with cold PBS, resuspended in 1 ml of propidium iodide (PI) staining solution (50 µg/ml PI, 10 mg/ml RNAse A, 0.1% Triton X-100, and 0.1% sodium citrate in PBS), and set at room temperature for 30 min. The stained cells were analyzed on a FACSCalibur flow cytometer for relative DNA content. The resulting data was evaluated using the FlowJo software (Tree Star, Inc., Ashland, OR).

### Statistic Analysis of Gene Expression Data

mRNA expression quantification data was analyzed by one-way analysis of variance (ANOVA) followed by Fisher's exact test. A *p*-value less than 0.05 was considered statistically significant.

## Supporting Information

Table S1 Sequences for dsRNAs and oligonucleotide primers.
(0.02 MB PDF)

Figure S1 Induction of E-cadherin and p21 by RNAa inhibits colony formation of COS1 and WES cells. COS1 and WES cells were transfected with 25 nM of the indicated saRNAs. Mock treatments were transfected in the absence of saRNA. One day after transfection, cells were seeded in 6-well plates at a density of 2,000 cells/well. Colony formation was analyzed at day 12 by staining the cells with crystal violet.
(0.12 MB JPG)

Figure S2 VEGF saRNA transfection increases levels of secreted VEGF. COS1 and WES cells were seeded in 12-well plates and transfected at with 25 nM concentrations of saRNA. Culture medium was collected and relative VEGF (VEGF_165_) concentrations were determined by ELISA. VEGF concentrations are shown as fold induction relative to mock treatments. Results are presented as mean ± SEM of two independent experiments.
(0.12 MB JPG)

Figure S3 NKX3-1 saRNA inhibits COS1 and WES cell growth. A. COS1 and WES cells were transfected with 25 nM of the indicated dsRNAs for 96 hrs. Cell images were taken at 100× magnification by phase contrast microscopy. Note: dsNKX3-1-381 transfected cells appear less dense and have acquired narrower, elongated shapes compared to control treatments. B. Cell density was quantified by counting the number of attached cells from five randomly selected fields as viewed under an inverted microscope at 100× magnification. Cell density and statistical significance (** p<0.001) is shown relative to mock transfections.
(0.78 MB JPG)
