# Supplementary material for: RNAa Is Conserved in Mammalian Cells
Source: PLoS One. 2010 Jan 22;5(1):e8848. doi: 10.1371/journal.pone.0008848 (PMC2809750; doi:10.1371/journal.pone.0008848)
Supplement: Table S1 — Sequences for dsRNAs and oligonucleotide primers. (0.02 MB PDF) [file pone.0008848.s001.pdf]

Supplementary Table. Sequences for dsRNAs and oligonucleotide primers

dsRNA name*	Sequence (5' - 3')
dsEcad-215 S	AAC CGU GCA GGU CCC AUA A[dT][dT]
dsEcad-215 AS	UUA UGG GAC CUG CAC GGU U[dT][dT]
dsP21-322 S	CCA ACU CAU UCU CCA AGU A[dT][dT]
dsP21-322 AS	UAC UUG GAG AAU GAG UUG G[dT][dT]
dsControl S	ACU ACU GAG UGA CAG UAG A[dT][dT]
dsControl AS	UCU ACU GUC ACU CAG UAG U[dT][dT]
dsP53-285 S	UUA CGG AAA GCC UUC CUA A[dT][dT]
dsP53-285 AS	UUA GGA AGG CUU UCC GUA A[dT][dT]
dsVEGF-706 S	GCA ACU CCA GUC CCA AAU A[dT][dT]
dsVEGF-706 AS	UAU UUG GGA CUG GAG UUG C[dT][dT]
dsVEGF-359 S	CCU UCA AUA UUC CUA GCA A[dT][dT]
dsVEGF-359 AS	UUG CUA GGA AUA UUG AAG G[dT][dT]
dsWT1-319 S	GAC UCA CUG CUU ACC UGA A[dT][dT]
dsWT1-319 AS	UUC AGG UAA GCA GUG AGU C[dT][dT]
dsPAR4-510 S	GUC UAG UCA UUC CCU GAA U[dT][dT]
dsPAR4-510 AS	AUU CAG GGA AUG ACU AGA C[dT][dT]
dsNKX3-1-360 S	GAC UGU UUG UCU UGA UCG U[dT][dT]
dsNKX3-1-360 AS	ACG AUC AAG ACA AAC AGU C[dT][dT]
dsNKX3-1-360-PT S	GAC UGU UUG UCU UGA UCG U[dT][dT]
dsNKX3-1-360-PT AS	ACG AUC AAG ACA AAC AGU C[dT][dT]
dsNKX3-1-360-CA S	GAG UGU UCG UCU UGG UCA U[dT][dT]
dsNKX3-1-360-CA AS	AUG ACC AAG ACG AAC ACU C[dT][dT]
dsNKX3-1-381 S	GAC GGU CCU GAA GAG CUA A[dT][dT]
dsNKX3-1-381 AS	UUA GCU CUU CAG GAC CGU C[dT][dT]
dsRB1-239 S	CCA CCA GAC UCU UUG UAU A[dT][dT]
dsRB1-239 AS	UAU ACA AAG AGU CUG GUG G[dT][dT]
dsVDR-267 S	GUA UCC GCA CCU AUA AUC A[dT][dT]

dsVDR-267 AS	UGA UUA UAG GUG CGG AUA C[dT][dT]
dsIL2-248 S	UUA GCU CAU UGU GUG GAU A[dT][dT]
dsIL2-248 AS	UAU CCA CAC AAU GAG CUA A[dT][dT]
dsPS2-136 S	CUA GGA AAC ACC UUU GAU A[dT][dT]
dsPS2-136 AS	UAU CAA AGG UGU UUC CUA G[dT][dT]
dsP27-48 S	CUC CUC CUC UGU UUA AAU A[dT][dT]
dsP27-48 AS	UAU UUA AAC AGA GGA GGA G[dT][dT]
dsCcnb1-597 S	AGA GAA ACC CUG UCU CGA A[dT][dT]
dsCcnb1-597 AS	UUC GAG ACA GGG UUU CUC U[dT][dT]
dsCcnb1-313 S	UAG CUU GGA CAG CAC ACA A[dT][dT]
dsCcnb1-313 AS	UUG UGU GCU GUC CAA GCU A[dT][dT]
dsCxcr4-467 S	cug aug uca uua uug uga a [dT][dT]
dsCxcr4-467 AS	uuc aca aua aug aca uca g [dT][dT]
dsCxcr4-438 S	cac aca cuu gcu ugg aug a [dT][dT]
dsCxcr4-438 AS	uca ucc aag caa gug ugu g [dT][dT]
dsCxcr4-359 S	uug ccg ccu acu ggu uag g [dT][dT]
dsCxcr4-359 AS	ccu aac cag uag gcg gca a [dT][dT]
dsCxcr4-299 S	caa ggc cuc cag acc ccu a [dT][dT]
dsCxcr4-299 AS	uag ggg ucu gga ggc cuu g [dT][dT]
dsCxcr4-283 S	cua aac ucc agg ucu aga a [dT][dT]
dsCxcr4-283 AS	uuc uag acc ugg agu uua g [dT][dT]
***: number in dsRNA name denotes target location relative to transcription start site. PT: Pan troglodytes (Chimpanzee); CA: Cercopithecus aethiops (African green monkey)**	
**Primer name**	**Sequence (5' - 3')**
**RT-PCR** primers	
NKX3-1 S	CTGAGGCCTGGGAGTCTCTT
NKX3-1 AS	AGCCCAAACCACAGAAAATG
WT1 S	GACCACCTGAAGACCCACAC
WT1 AS	TGTGATGGCGGACTAATTCA

p53 S	CCCCTCTGAGTCAGGAAACA
p53 AS	TCATCTGGACCTGGGTCTTC
VEGF S	TCTTCAAGCCATCCTGTGTG
VEGF AS	CTATGTGCTGGCCTTGGTG
ACTB S	GCAAAGACCTGTACGCCAAC
ACTB AS	GTACTTGCGCTCAGGAGGAG
Ecad S	AAGAAGGAGGCGGAGAAGAG
Ecad AS	GGCTGTGGGGTCAGTATCAG
p21 S	GGAAGACCATGTGGACCTGT
p21 AS	GGATTAGGGCTTCCTCTTGG
PAR4 S	AGGGATGCAAATGTTTCAGG
PAR4 AS	TCCTGCTTTAGCTGTTCATTTTC
pS2 S	TTGTGGTTTTCCTGGTGTCA
pS2 AS	GCAGATCCCTGCAGAAGTGT
VDR S	GTTCCAGGTGGGACTGAAGA
VDR AS	RACGTCTGCAGTGTGTTGGAC
IL2 S	CAAACCTCTGGAGGAAGTGC
IL2 AS	ATGGTTGCTGTCTCATCAGC
RB1 S	GGAAGCAACCCTCCTAAACC
RB1 AS	TTTCTGCTTTTGCATTCGTG
Ccnb1 S	ctccctgcttcctgttatgc
Ccnb1 AS	ttcgacaacttccgttagcc
rCXCR4 S	ACTTCAACAGGATCTTCCTGCCCA
rCXCR4 AS	TGGAGTGTGACAGCTTGGAGATGA
rβ-Actin S	TCTACAATGAGCTGCGTGTG
rb-Actin AS	AATGTCACGCACGATTTCCC
**Promoter DNA PCR primers**	
p21-pF1	GCTGCATTGGGTAAATCCTT
p21-pR1	AAGGAACTGACTTCGGCAGC
Ecad-pF1	GTGAGCCCCATCTCCAAAAC
Ecad-pR1	CTTTGCAGTTCCGACGCCAC

p53-CF1	CGCAGCAGGTCTTGCACCTCT
p53-CR2	TTTGCGTTTGCTCTCAGCTGG
VEGF-CF1	GGCCAGATGAGGGCTCCAGAT
VEGF-CR1	CTAGTGACTGCCGTCTGCACAC
VEGF-CF2	GGATTCCAATAGATCTGTGTGTC
VEGF-CR2	CGGAAACTCTGTCCAGAGAC
PAR4-cF1	CTCCCGCAACCCTAAGATAC
PAR4-cR1	CCACCACTCTTCCTTTTAAG
WT1-cF1	TCTGCGCTTTCCTGAAGTTC
WT1-cR1	GACACCCTCCTCTTCAACCC
NKX3-1-cF1	GGCTGCAGTGACTGCGTGCTC
NKX3-1-cR1	TCCGCCCTGAGCCGTTTTCC
