# Supplementary figures and images for: RNAa Is Conserved in Mammalian Cells
Source: PLoS One. 2010 Jan 22;5(1):e8848. doi: 10.1371/journal.pone.0008848 (PMC2809750; doi:10.1371/journal.pone.0008848)

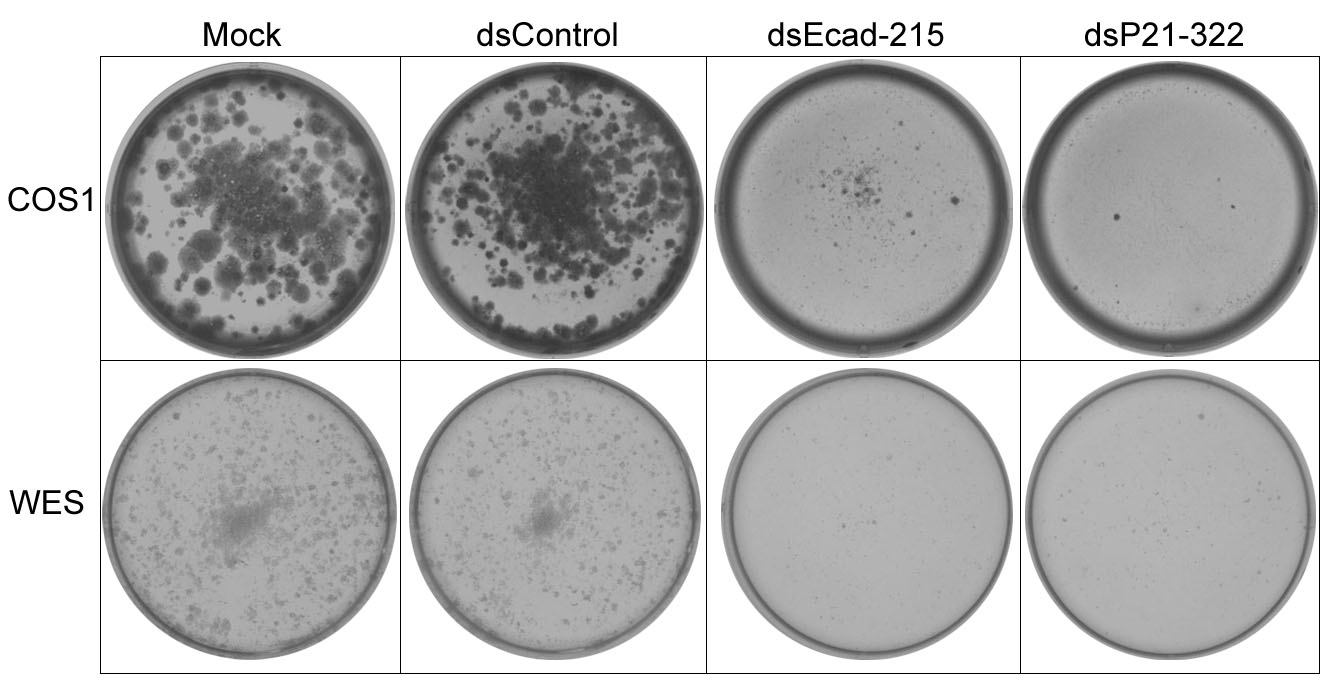

Supplement: Figure S1 — Induction of E-cadherin and p21 by RNAa inhibits colony formation of COS1 and WES cells. COS1 and WES cells were transfected with 25 nM of the indicated saRNAs. Mock treatments were transfected in the absence of saRNA. One day after transfection, cells were seeded in 6-well plates at a density of 2,000 cells/well. Colony formation was analyzed at day 12 by staining the cells with crystal violet. (0.12 MB JPG) [file pone.0008848.s002.jpg]

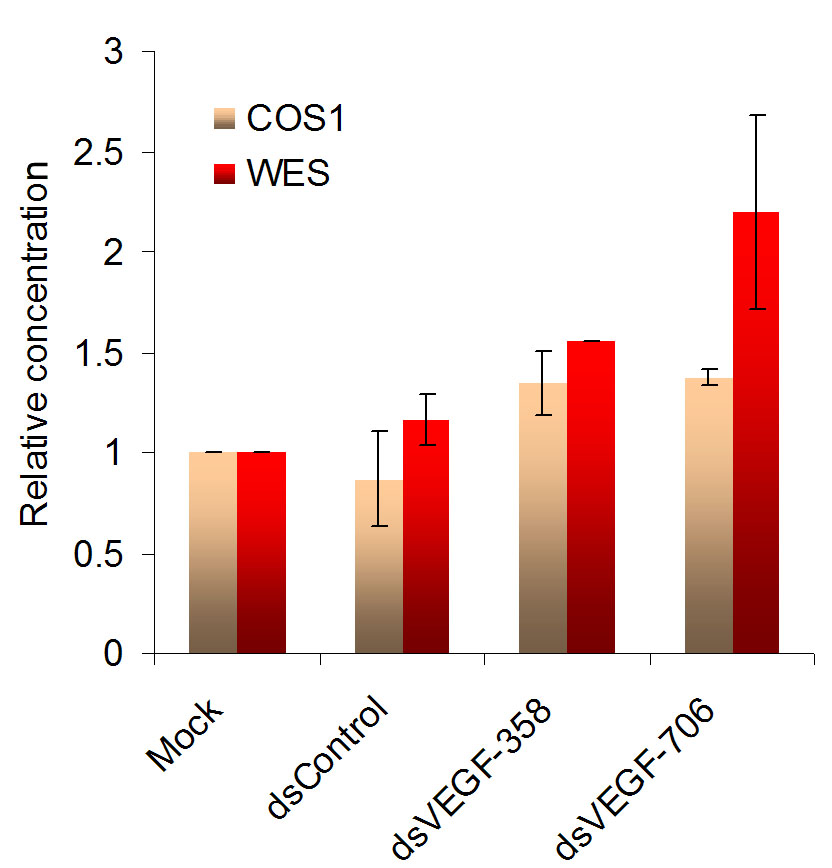

Supplement: Figure S2 — VEGF saRNA transfection increases levels of secreted VEGF. COS1 and WES cells were seeded in 12-well plates and transfected at with 25 nM concentrations of saRNA. Culture medium was collected and relative VEGF (VEGF165) concentrations were determined by ELISA. VEGF concentrations are shown as fold induction relative to mock treatments. Results are presented as mean ± SEM of two independent experiments. (0.12 MB JPG) [file pone.0008848.s003.jpg]

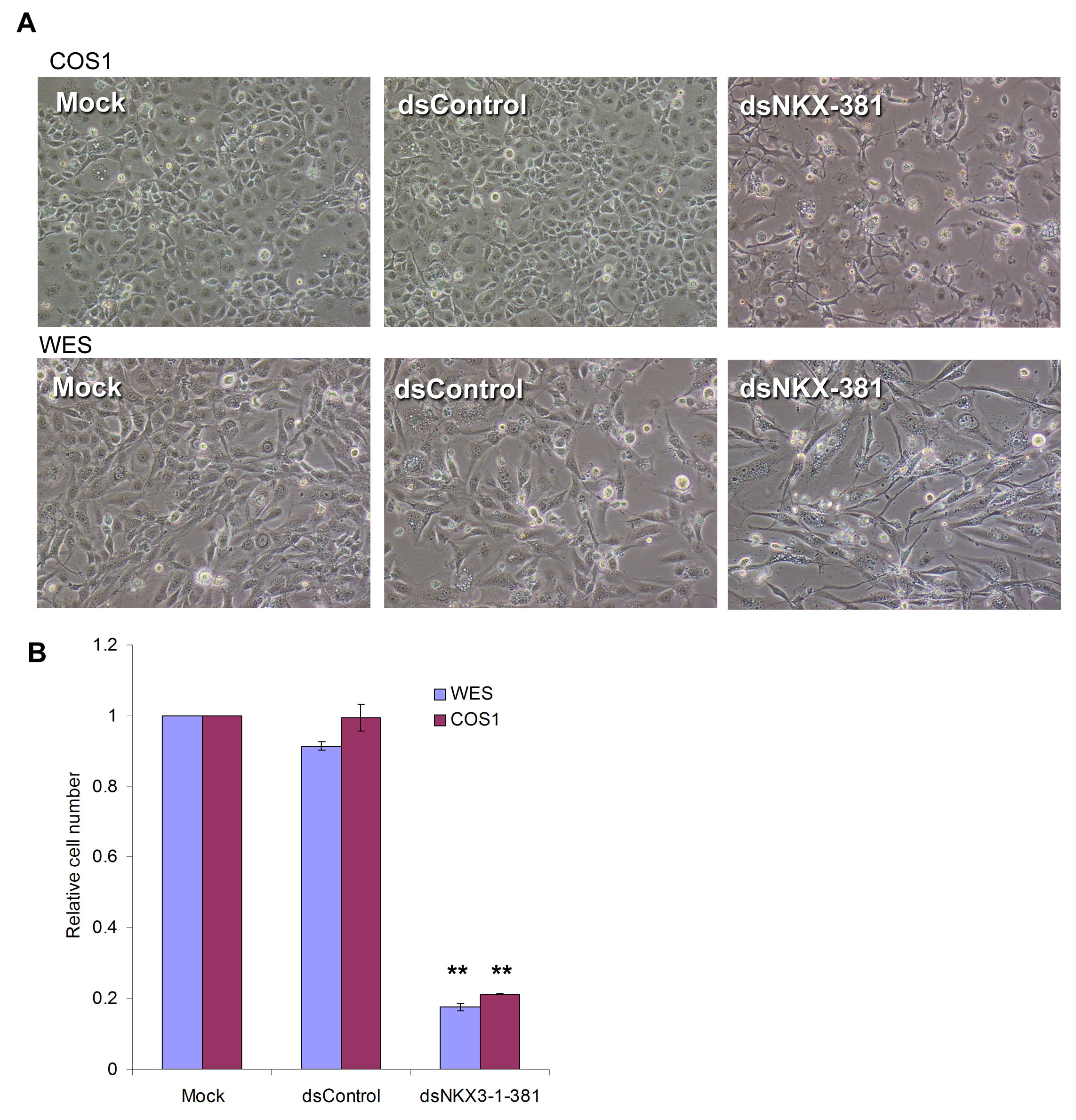

Supplement: Figure S3 — NKX3-1 saRNA inhibits COS1 and WES cell growth. A. COS1 and WES cells were transfected with 25 nM of the indicated dsRNAs for 96 hrs. Cell images were taken at 100× magnification by phase contrast microscopy. Note: dsNKX3-1-381 transfected cells appear less dense and have acquired narrower, elongated shapes compared to control treatments. B. Cell density was quantified by counting the number of attached cells from five randomly selected fields as viewed under an inverted microscope at 100× magnification. Cell density and statistical significance (** p<0.001) is shown relative to mock transfections. (0.78 MB JPG) [file pone.0008848.s004.jpg]
